# Gamma-delta (γδ) T-cell lymphoma – another case unclassifiable by World Health Organization classification: a case report

**DOI:** 10.1186/s13256-017-1312-5

**Published:** 2017-06-19

**Authors:** Hemant Sindhu, Ruqin Chen, Hui Chen, Jonathan Wong, Rashid Chaudhry, Yin Xu, Jen C. Wang

**Affiliations:** 1 Division of Hematology/Oncology, Brookdale University Hospital Medical Center, Brooklyn, NY 11212 USA; 2 Department of Surgery, Brookdale University Hospital Medical Center, Brooklyn, NY USA; 3 Genoptix Medical Laboratory, Carlsbad, CA USA

**Keywords:** Gamma-delta T-cell lymphoma (Tγδ cell lymphoma), T-cell lymphoma NOS, Case report, Hepatosplenic Tγδ, Cutaneous Tγδ

## Abstract

**Background:**

We present a case of gamma-delta T-cell lymphoma that does not fit the current World Health Organization classifications.

**Case presentation:**

A 74-year-old Caribbean-American woman presented with lymphocytosis, pruritus, and non-drenching night sweats. Bone marrow and peripheral blood analyses both confirmed the diagnosis of gamma-delta T-cell lymphoma. An axillary lymph node biopsy was negative for lymphoma. Clinically absent hepatosplenomegaly and skin lesions with biopsy-proven gamma-delta T-cell lymphoma suggest that she is unclassifiable within the current classification system.

**Conclusions:**

We believe this is a case of not otherwise specified gamma-delta T-cell lymphoma. Accumulation of these rare not otherwise specified cases will be important for future classification which further defines the biology of this disease.

## Background

T lymphocytes recognize antigens through T cell receptors (TCRs). TCRs are polypeptide heterodimers which are commonly made up of α and β chains and rarely of γ and δ chains. Gamma-delta T cells (Tγδ) thus represent less than 5% of all peripherally circulating lymphocytes and of all T cells in mucosa, these cells can account for up to 50%. Tγδ have a selective tropism for the red pulp of the spleen, mucosal tissues, and lymphoid tissues, as well as gastrointestinal epithelial tissues and skin [1]. The majority of the lymphoid tissues, however, are distributed by the alpha-beta T cell. Therefore, the alpha-beta receptor T cells are expressed in most T-cell lymphomas (TCLs), and gamma-delta TCR is expressed by less than 5% of TCL. Tγδ cell lymphoma represents a rare and aggressive set of neoplasms which portend a very poor prognosis [2]. In 2008, the World Health Organization (WHO) redefined its classification of Tγδ cell lymphoma into two groups: hepatosplenic Tγδ cell lymphoma (HSγδTL) and primary cutaneous TCL (PCTCL) [3]. Tripodo *et al*. asserted that the 2008 WHO classification accounts for the cellular, phenotypic, and molecular properties of hematopoietic and lymphoid tissues in this updated classification [4]. In 2016, the WHO classification of T Tγδ cell lymphoma did not add much [5]. Although the WHO classification is helpful in defining the subtypes of lymphomas, the rare entities comprising peripheral TCLs (PTCLs) remain less well understood given their low incidence as well as the nebulous nature of the T cell system [6]. HSγδTL is most commonly seen in young immunocompromised men aged 20 to 25 years. Variations in age of presentation are possible with Crohn’s disease where younger patients including children are afflicted. Recipients of solid-organ transplants are more likely to present at a later age. PCTCL does not show a gender predisposition, but is described in older patients with a median age of 60 years [7]. PCTCL occurs in less than 1% of cases of primary cutaneous Tγδ cell lymphoma; those with cutaneous involvement demonstrate a poor prognosis. Here we describe a case of a 74-year-old woman with biopsy-proven Tγδ cell lymphoma that fits neither of the two classifications of Tγδ cell lymphoma because she does not have hepatosplenomegaly and neither does she exhibit the typical cutaneous manifestations. This report adds to the database of these atypical cases of Tγδ cell lymphoma not classified by the current WHO criteria.

## Case presentation

A 74-year-old Caribbean-American woman was referred by her primary care physician for evaluation of lymphocytosis. She complained of generalized itching and non-drenching night sweats, and denied any unexpected weight loss. Her past medical history is significant for breast cancer treated with mastectomy in 2001. At the time of presentation, she was not taking any medication and had no history of tobacco smoking or recreational substance use. She is a homemaker and immigrant whose only positive family history is breast cancer in her mother diagnosed at age 80 years. Her physical examination was grossly normal revealing no cutaneous lesions and no hepatosplenomegaly, but there were two palpable axillary lymph nodes measuring 2 to 3 cm.

**Fig. 1:**
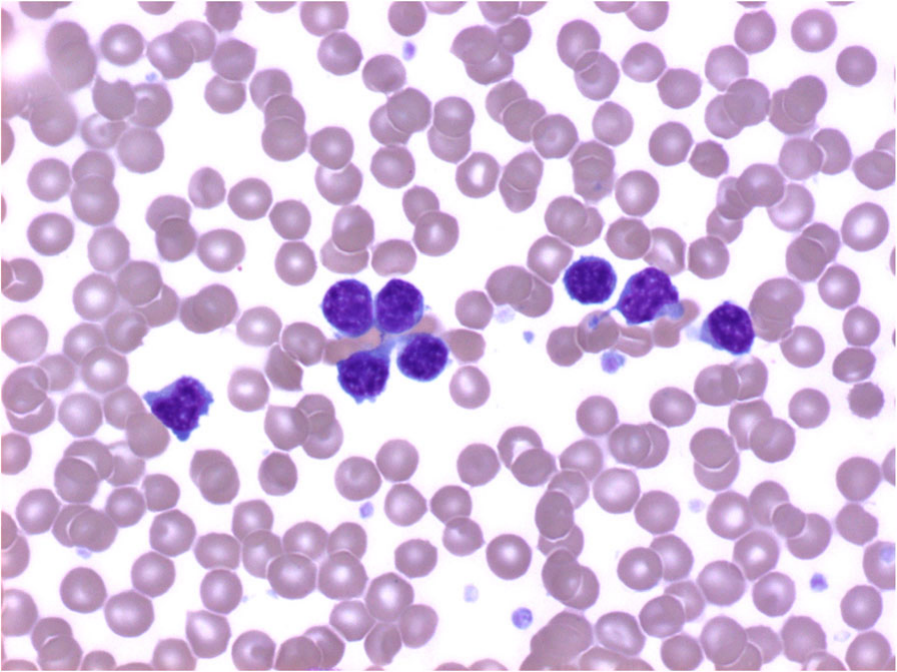
Atypical lymphocytosis. The blood smear shows increased small-sized to medium-sized lymphocytes with mature chromatin

**Fig. 2:**
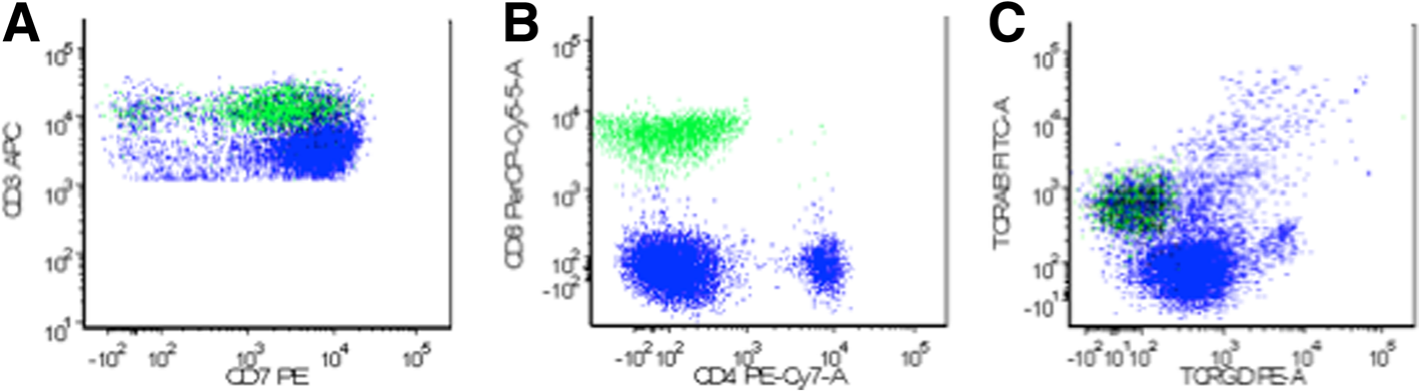
Identification of aberrant gamma-delta T cells by flow cytometry. **a** A predominant population of T cells with dim CD3 expression. **b** The CD3 dim-positive T cells are dual negative for CD4 and CD8. **c** The CD3 dim-positive T cells are positive for T cell receptor gamma-delta

**Fig. 3:**
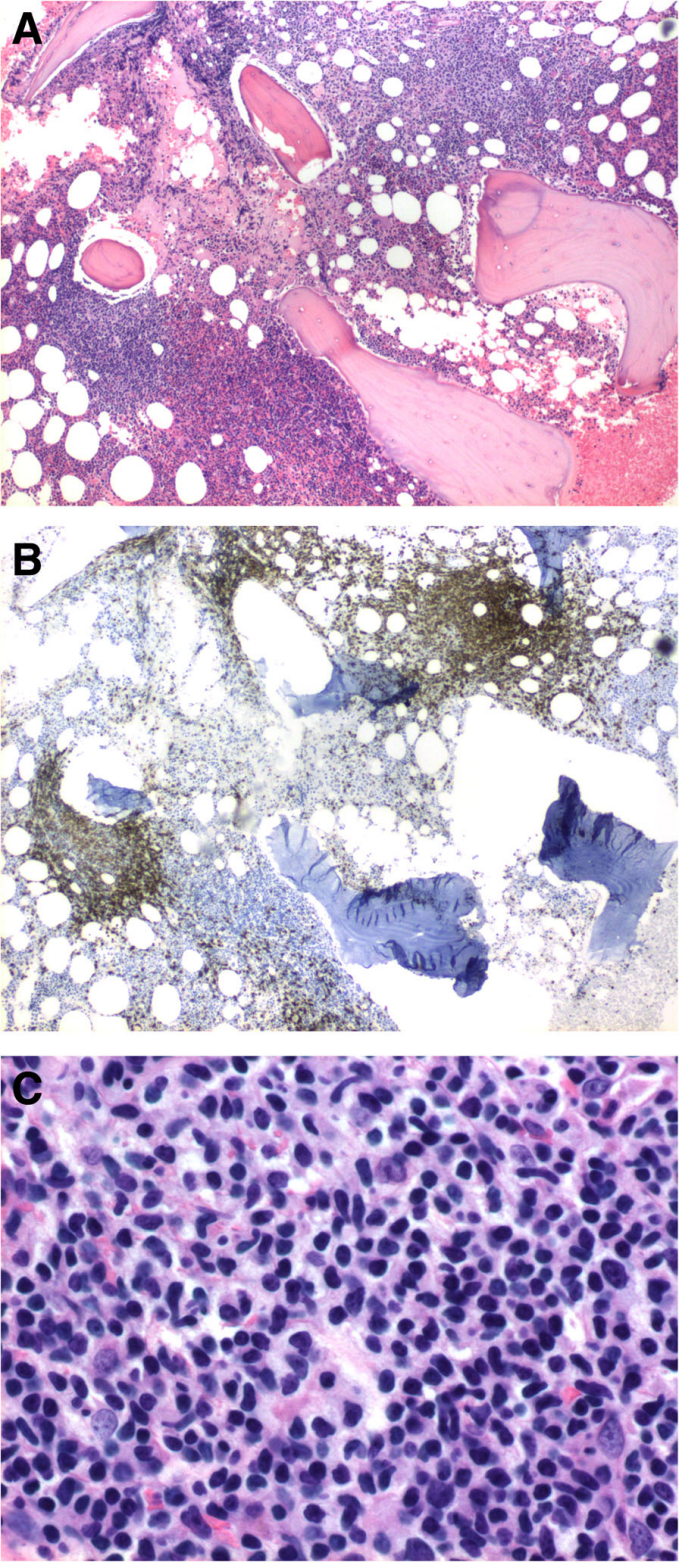
Bone marrow involvement by T-cell lymphoma. **a** Paratrabecular and interstitial lymphoid aggregates. **b** CD3 immunostaining highlights T cells in the aggregates. **c** Small-sized to medium-sized atypical lymphocytes with mature chromatin

Her white cell count was 11.3×10^9^ cells/L, with a prominent absolute lymphocyte count (7.5×10^9^/L) and increased atypical mature lymphocyte (Fig. 1). She also had elevated levels of creatinine (1.3 mg/dL), immunoglobulin G (IgG) (2037 mg/dL), and lactate dehydrogenase (397 IU/L). Her transaminases and tests of liver function were unremarkable. A computed tomography (CT) scan revealed axillary and abdominal portacaval lymphadenopathy. A positron emission tomography (PET)-CT scan showed fluorodeoxyglucose (FDG) avidity in her left axillary and inferior portacaval nodes as well as the bone marrow. Flow cytometry of her peripheral blood revealed 37% aberrant Tγδ, which were positive for CD2, CD3(dim), CD5, CD7, TCR gamma-delta, and negative for CD1a, CD4, CD8, CD10, CD16, CD25, CD56, CD57, terminal deoxynucleotidyl transferase (TdT), and TCR alpha-beta (Fig. 2). Molecular analysis also demonstrated TCRs with gamma-delta rearrangements, which supported a mature T (γδ) clonal proliferative disease. A subsequently performed bone marrow biopsy revealed both paratrabecular and interstitial aggregates of small-sized to medium-sized lymphocytes which stained predominantly positive for T cells (Fig. 3a–c). Immunohistochemistry showed that the CD3/CD5-positive T cells were negative for both CD4 and CD8 as well as TIA-1 and Granzyme B. Flow cytometry of the bone marrow aspirate detected 26% TCR Tγδ with the same immunophenotype as seen in the blood. Together, the bone marrow findings were consistent with marrow involvement by a mature gamma-delta T cell neoplasm. The lymph node biopsy showed preserved nodal architecture with many lymphoid follicles. Since the lymph node architecture was preserved, it was probable that the lymph nodes were not involved in the pathology. This diagnosis was made after consultation with Dr Elaine Jaffe at the National Institutes of Health (NIH). Cytogenetics was normal in the bone marrow as well as in the lymph node biopsies. Fluorescence *in situ* hybridization (FISH) utilized probes specific for T cell neoplasm including 1p/1q, 5/5q, 7/7q, centromere 8, and 14q11 did not detect any aberrations in all 200 nuclei examined. Our patient was started on cyclophosphamide, vincristine, doxorubicin, and prednisone (CHOP). She experienced grade 4 nausea and vomiting and was hospitalized twice with febrile neutropenia. Through the course of treatment, step-wise dose reductions were made due to intolerability. An interval PET-CT in December 2015 revealed a favorable radiographic and scintigraphic response to treatment. Specifically, there was resolution of previously identified lymphadenopathy below her diaphragm. A mildly FDG-avid slightly enlarged level I lymph node was present in the right axilla. There was FDG accumulation throughout the bone marrow suggestive of treatment-related hyperplasia, but no definite evidence of focal areas of more pronounced FDG accumulation was present within her osseous structures, which showed resolution. She received a total of eight cycles of CHOP, the final cycle being dose-reduced by 70%. In March 2016, PET-CT revealed recurrence of hypermetabolic lymphadenopathy in her right axillary, supraclavicular, and left hilar regions. Her symptoms of itching and night sweats also returned. She refused any further intervention. She subsequently developed emboli in her feet bilaterally without an evident source. There were no atrial or valvular vegetations visible on transthoracic echocardiography. She underwent bilateral below-the-knee amputations and she eventually succumbed to pneumonia postoperatively in 2016; she survived approximately 16 months post-diagnosis.

## Discussion

It is the taxonomy and classification of diseases that form the linguistics of medicine. Hence we rely on our guidelines and cataloging to aid us in diagnosis and guide us with treating our patients. Just as our knowledge evolves, so must our system of categorizing. In this 2008 WHO update, as published in the fourth edition of the *Classification of Tumours of Haematopoietic and Lymphoid Tissues*, which built on the third edition published in 2001, precursor lesions of lymphoid neoplasms earned more notoriety through sub-classification [3]. It is in fact the Revised European-American Lymphoma (REAL) classification which provides the foundation for the WHO system [8]. Although the WHO classification is helpful in defining the subtypes of lymphomas, the rare entities comprising PTCLs remain less well understood given their low incidence as well as the nebulous nature of the T cell system [9]. Further down the taxonomic framework of PTCLs, Tγδ cell lymphomas were disambiguated into two main entities: hepatosplenic TCL (HSTL) and primary cutaneous gamma-delta TCL (PCγδ-TCL).

Tγδ cell lymphomas are neoplasms which arise from a very small subset of T lymphocytes expressing gamma-delta TCRs. Similar to their B cell counterparts, T lymphocytes use these receptors for cell or antigen recognition; the receptor itself a heterodimer which comprises either the more common alpha-beta combination or, less commonly, the gamma-delta amalgamation [10]. The gamma-delta cells human γδ T lymphocytes originate from CD4-CD8- double negative progenitor cells [11]. The gamma-delta T cell population is further divided based on differences in the variable receptor region: Vdelta1 and Vdelta2 [4]. These two subpopulations are not only distinct in their phenotypic variance, but also by their distribution as Vdelta1 cells are most commonly found in the gastrointestinal tract whereas Vdelta2 cells have a preponderance for the skin and lymphoid tissue [12]. Of the two subtypes of gamma-delta TCLs, HSTL was the first to be described in the literature; HSTL is classically described as having the gamma-delta phenotype (preferentially derived from Vdelta1 type) with only rarely described cases harboring the alpha-beta subtype [13]. Clinically, HSTL is a disease primarily described in young men, especially in those who have been exposed to chronic immunosuppressants [12]. In addition to young adults having the risk factor of chronic inflammation such as Crohn’s disease, it has been described in the setting of those individuals with chronic hepatitis B infection [14]. The most common physical finding is splenomegaly, and hepatomegaly is noted in nearly 90% of cases whereas there is a complete lack of lymphadenopathy. Symptoms that often bring individuals to the clinical setting are B symptoms, early satiety, abdominal pain, and weakness [15]. Laboratory data usually reflect transaminitis, and elevations in alkaline phosphatase and lactate dehydrogenase. Cytopenias are commonly seen, thrombocytopenia being the most common (85% of cases) followed by anemia (75% of cases) [16]. The mucocutaneous gamma-delta TCLs can be divided into those localized to the skin and those involving the nasal cavity, the bowel, the respiratory tract, and the thyroid. Both these entities, however, preferentially derive from the Vdelta2 cells subset and tend to have an activated cytotoxic profile (TIA1+, Granzyme B+, Perforin+) [12].

Based on laboratory data including findings on bone marrow biopsy and peripheral blood findings, our patient has a definite diagnosis of a mature gamma-delta TCL. However, her clinical findings are not consistent with either of the two WHO classifications given that she has no hepatosplenomegaly and lacks the typical cutaneous manifestations. Moreover, our 74-year-old patient fits neither age range of the two subtypes of γδ-TCL. The hepatosplenic variant has been better studied and as per previous reports, the median age of hepatosplenic γδ-TCL is less than 40 years, with the oldest reported age of 64 years. It is further reported that approximately 10 to 20% of patients have a prior history of immunocompromise, which is not present in our patient. Our patient’s lack of the expression of Granzyme B+ and Perforin staining coupled with the absence of skin involvement makes the diagnosis of PCγδ-TCL unlikely. The lack of overt lymph node involvement in our patient is also described in cases of HSTL and PCγδ-TCL [12]. For these patients with Tγδ cell lymphoma, the median survival reported is 1 to 2 years and there is no standard treatment which has proven to be effective. Those patients treated with chemotherapy tended toward a longer survival. There is a single report of a patient treated with allogeneic stem cell transplant which revealed a survival longer than 7 years. Data suggest that women have longer overall survival compared to men (25 months versus 8 months) [17, 18].

## Conclusions

Our patient is among the first of such cases described in the literature whose age, presentation, and laboratory findings do not fit neatly into either of the two WHO classifications of γδ-TCL. Since many of these rare cases of Tγδ cell lymphoma remain unclassifiable, a literary accumulation of these reports will be important for future classification.

## Abbreviations

CHOP: Cyclophosphamide, vincristine, doxorubicin, and prednisone
CT: Computed tomography
FDG: Fluorodeoxyglucose
HSγδTL: Hepatosplenic Tγδ cell lymphoma
HSTL: Hepatosplenic T-cell lymphoma
NOS: Not otherwise specified
PCγδ-TCL: Primary cutaneous gamma-delta T-cell lymphoma
PCTCL: Primary cutaneous T-cell lymphoma
PET: Positron emission tomography
PTCL: Peripheral T-cell lymphoma
TCL: T-cell lymphoma
TCR: T cell receptor
Tγδ: Gamma-delta T cells
WHO: World Health Organization
